# Persistent contamination of raw milk by *Campylobacter jejuni* ST-883

**DOI:** 10.1371/journal.pone.0231810

**Published:** 2020-04-21

**Authors:** Anniina Jaakkonen, Rauni Kivistö, Maria Aarnio, Jenni Kalekivi, Marjaana Hakkinen

**Affiliations:** 1 Microbiology Unit, Laboratory and Research Division, Finnish Food Authority, Helsinki, Finland; 2 Department of Food Hygiene and Environmental Health, Faculty of Veterinary Medicine, University of Helsinki, Helsinki, Finland; USDA-ARS Eastern Regional Research Center, UNITED STATES

## Abstract

*Campylobacter jejuni* has caused several campylobacteriosis outbreaks via raw milk consumption. This study reports follow-up of a milk-borne campylobacteriosis outbreak that revealed persistent *C*. *jejuni* contamination of bulk tank milk for seven months or longer. Only the outbreak-causing strain, representing sequence type (ST) 883, was isolated from milk, although other *C*. *jejuni* STs were also isolated from the farm. We hypothesized that the outbreak strain harbors features that aid its environmental transmission or survival in milk. To identify such phenotypic features, the outbreak strain was characterized for survival in refrigerated raw milk and in aerobic broth culture by plate counting and for biofilm formation on microplates by crystal violet staining and quantification. Furthermore, whole-genome sequences were studied for such genotypic features. For comparison, we characterized isolates representing other STs from the same farm and an ST-883 isolate that persisted on another dairy farm, but was not isolated from bulk tank milk. With high inocula (10^5^ CFU/ml), ST-883 strains survived in refrigerated raw milk longer (4–6 days) than the other strains (≤3 days), but the outbreak strain showed no outperformance among ST-883 strains. This suggests that ST-883 strains may share features that aid their survival in milk, but other mechanisms are required for persistence in milk. No correlation was observed between survival in refrigerated milk and aerotolerance. The outbreak strain formed a biofilm, offering a potential explanation for persistence in milk. Whether biofilm formation was affected by pTet-like genomic element and phase-variable genes encoding capsular methyltransferase and cytochrome C551 peroxidase warrants further study. This study suggests a phenotypic target candidate for interventions and genetic markers for the phenotype, which should be investigated further with the final aim of developing control strategies against *C*. *jejuni* infections.

## Introduction

*Campylobacter jejuni*, which is the leading cause of bacterial gastroenteritis worldwide, is asymptomatically carried in the digestive tract of numerous wild and domesticated bird and mammal species. Human infection is usually acquired by the consumption of contaminated poultry meat, water, raw milk, or contact with animal feces. *C*. *jejuni* is prevalent in cattle and the consumption of raw cow’s milk has mediated several campylobacteriosis outbreaks [1].

*C*. *jejuni* grows optimally at 37‒42°C under microaerobic conditions and cannot tolerate drying and atmospheric levels of oxygen. Despite fastidious growth requirements, *C*. *jejuni* possesses mechanisms to survive in stress conditions, which play a role in host colonization, transmission in the environment, and survival in the food chain to cause human infection [2–4]. Such mechanisms include defense against atmospheric levels of oxygen and reactive oxygen species, heat shock, low pH, osmotic stress, and nutrient-poor environments. *C*. *jejuni* can survive, but not proliferate, in nutrient-poor cold waters for months [5].

One strategy for survival in harsh conditions is biofilm formation. *C*. *jejuni* can form biofilm on a variety of abiotic surfaces and coexist with other species in polymicrobial biofilms [6]. Indeed, secondary colonization of existing biofilms by *C*. *jejuni* has been suggested to occur on poultry farms [7]. *C*. *jejuni* biofilms have not been reported on dairy farms to our knowledge, but milking equipment could potentially allow biofilm formation like other food production and processing environments.

Biofilm formation by *C*. *jejuni* is a complex process involving several gene functions, not yet fully elucidated. As suggested, genetic mechanisms behind the biofilm-forming phenotype may even vary between different *C*. *jejuni* lineages: ST-21 CC and ST-45 CC [8]. Biofilm formation has been associated with surface proteins, flagella, and quorum sensing in mutational studies. Furthermore, shifted expression levels have been observed in biofilm-grown *C*. *jejuni* towards iron uptake, oxidative stress defense, and membrane transport [3].

Inter-strain variability has been observed both in the ability to cope with environmental stresses and in niche adaptation. As revealed by multilocus sequence typing (MLST), certain *C*. *jejuni* lineages (such as ST-21 CC) are found more often in human infection and in the food chain, whereas others (such as ST-45 CC) are often present in environmental sources with less clinical impact [3,4]. Furthermore, host specificity is common among lineages in wildlife, whereas lineages in livestock (such as ST-21 CC and ST-45 CC) show more generalist nature and often coexist in farm environments. A few livestock-associated host-specialist lineages are also known, such as ST-61 CC in cattle [9,10]. Better understanding of the survival strategies and transmission patterns of *C*. *jejuni* strains in farm environments is required to develop control strategies that would lessen the disease burden of this pathogen.

In 2012, a follow-up study of a campylobacteriosis outbreak revealed persistent *C*. *jejuni* contamination in bulk tank milk on a Finnish dairy farm. Interestingly, only the outbreak strain was isolated from milk, although other strains were detected on the farm simultaneously. We hypothesized that the outbreak strain possesses survival mechanisms to aid its on-farm transmission or survival in milk. We studied the outbreak strain for survival in refrigerated raw milk, aerotolerance, biofilm formation, antimicrobial susceptibility, and genomic content to explore mechanisms behind persistence. Ultimately, we aimed to determine whether and, if so, why certain *C*. *jejuni* strains pose a higher health risk in milk production settings to aid the development of enhanced control strategies.

## Results

### *C*. *jejuni* in bulk tank milk and milk filters

Samples were collected at the dairy farm from bulk tank milk and milk filters 11 times during six months after the outbreak (December 2012 to June 2013). Simultaneously, rigorous hygienic measures were applied to eliminate *C*. *jejuni* contamination from the farm. Despite hygienic measures, *C*. *jejuni* was isolated from 10/11 milk samples (91%) and 10/21 milk filter samples (48%), being detected from milk or milk filters in all 11 samplings. The concentration of thermotolerant *Campylobacter* in the milk samples ranged from 0.007 to 35 MPN/ml. All *C*. *jejuni* isolates matched the outbreak type pattern in pulsed-field gel electrophoresis (PFGE) studies, suggesting that the strain persistently contaminated bulk tank milk for seven months or longer.

### *C*. *jejuni* in cattle feces and the farm environment

Cattle feces on the farm were sampled for *C*. *jejuni* twice within two months of the outbreak (in December 2012 and January 2013), and samples were collected from the farm environment throughout the six-month monitoring period. *C*. *jejuni* was isolated from 25/39 fecal samples (64%). Isolates from 12 fecal samples represented the outbreak type, while six other pulsotypes were detected from 13 fecal samples. Two cows carried the outbreak type in both samplings, whereas other pulsotypes were detected sporadically among cow specimens. *C*. *jejuni* of the outbreak type was isolated from only 3/54 environmental samples (5%) taken from the milk room and a feeding table, while a sporadic type was isolated from an udder cloth. Altogether, eight *C*. *jejuni* pulsotypes were detected on the farm, but interestingly only the outbreak type was isolated from bulk tank milk and milk filters and occurred repeatedly.

### Multilocus sequence typing of *C*. *jejuni* farm isolates

In seven-loci MLST, the outbreak type represented sequence type (ST) ST-883 and clonal complex (CC) ST-21 CC. Other farm pulsotypes represented were ST-45 (ST-45 CC), ST-50 (ST-21 CC), ST-58 (unassigned CC), and ST-61 (ST-61 CC). ST-50 was isolated from the udder cloth and the other three STs (ST-45, ST-58, and ST-61) from cattle.

Genomic epidemiology of ST-58 and ST-883 isolates was further studied using whole-genome multilocus sequence typing (wgMLST). Other STs were not studied because they were represented by only a single isolate. ST-58 farm isolates of this study (n = 3) were compared with ST-58 isolates from the UK (n = 34), representing publicly available ST-58 genomes. All ST-58 isolates were obtained from ruminant sources from 2011 to 2018, suggesting host-specificity. In the allelic profile size of 1064, two ST-58 farm isolates (ST58_2012–12_C1 and ST58_2013–01_C3) appeared within close pairwise distance (PWD) of 1 (0.1%). One farm isolate (ST58_2012–12_C2), however, appeared unrelated (PWD 4.7%), considering PWD of the closest UK isolate (5.5%) with the farm isolates of this study. Thus, two clones were recognized among the ST-58 isolates of this study.

**Fig 1:**
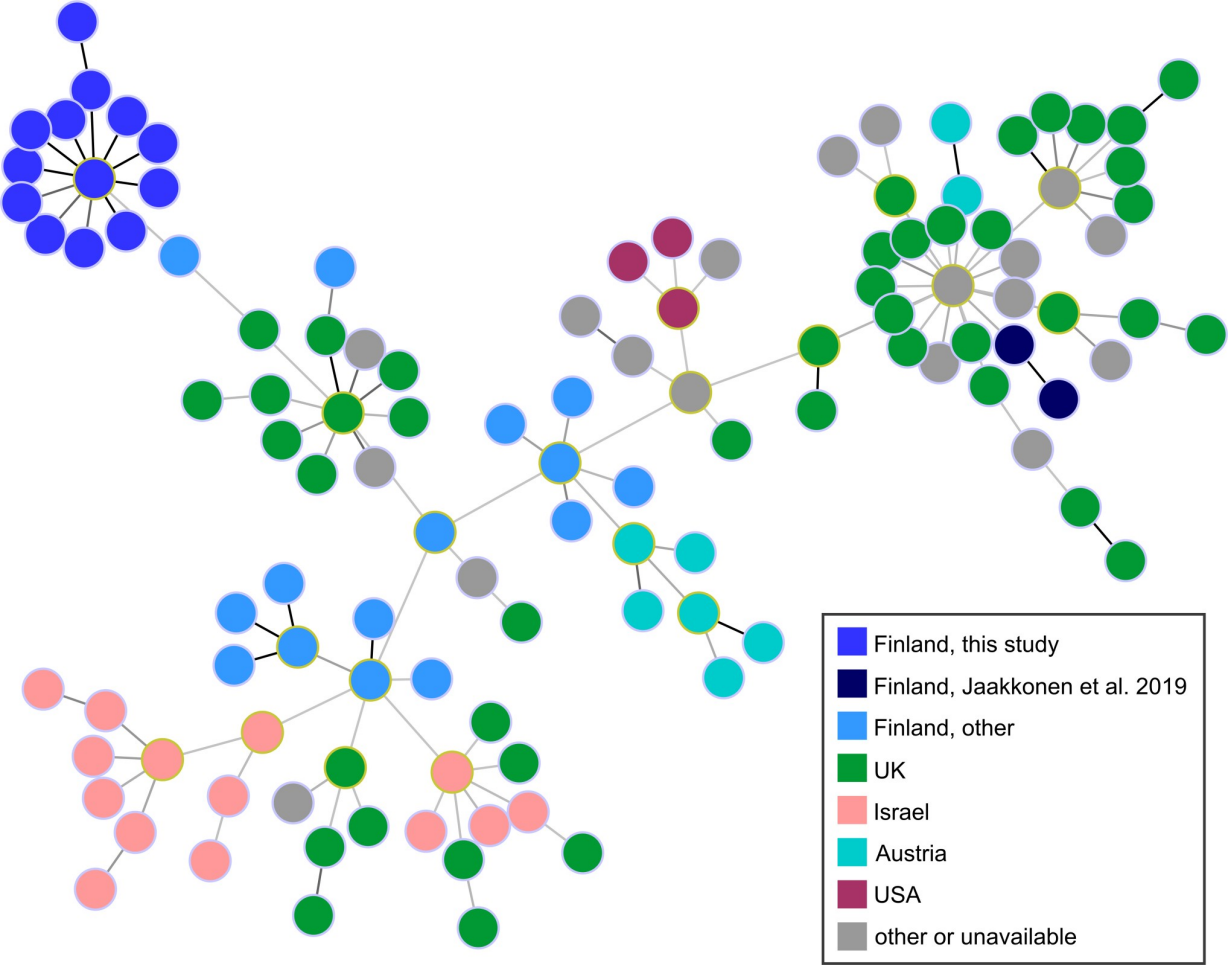
Minimum spanning tree from wgMLST comparison of ST-883 *C*. *jejuni* isolates. Dairy farm isolates from this study (n = 40) are compared with globally collected ST-883 isolates (n = 137), including dairy farm isolates from Jaakkonen et al. [11]. Nodes are colored by country. Black and dark gray links indicate short allelic distances of 1 and 2 loci, respectively, in the profile size of 718 loci.

ST-883 isolates of this study (n = 40) were compared with globally collected ST-883 isolates (n = 137). The dataset included ST-883 isolates (n = 5) that were found to persist on another Finnish dairy farm for 11 months or longer without being detected from bulk tank milk in weekly samplings [11]. In the allelic profile size of 718, the ST-883 isolates of this study appeared within the maximum PWD of 4 (0.6%). Closest outgroup isolate (IN_Cj_FI_109) appeared within PWD of 4.9% and was isolated from cattle in Finland in 2003. Isolates from the other Finnish dairy farm appeared within PWD of 21% from the isolates of this study. Finnish isolates were generally dispersed within the minimum spanning tree, showing no evidence of geographic circulation of the outbreak clone (Fig 1). When comparing only ST-883 isolates of this study, the maximum PWD was 5 (0.5%) in the allelic profile size of 1032. Therefore, ST-883 isolates of this study appeared similar in wgMLST, suggesting that the farm was contaminated by a single clone of ST-883, and the outbreak originated from the dairy farm. As revealed by MLST and further wgMLST analysis, altogether six clones were recognized among the isolates from the outbreak farm.

### Survival in refrigerated raw milk

**Fig 2:**
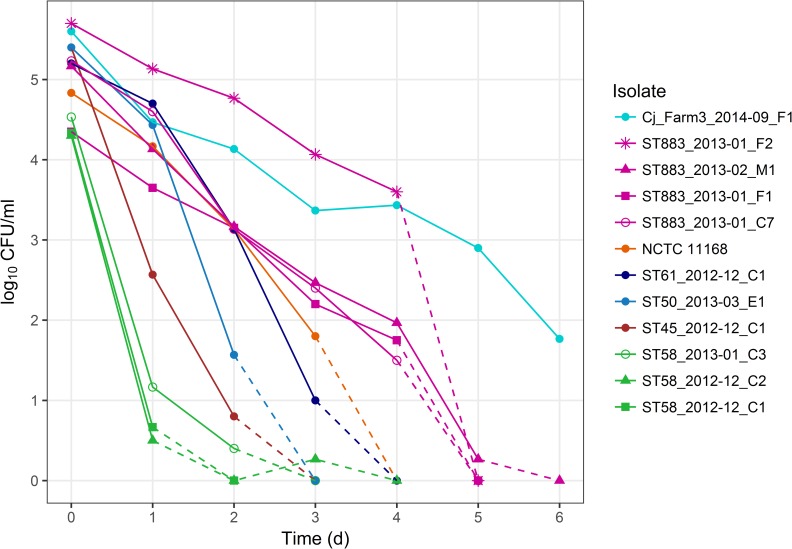
Survival of *C*. *jejuni* farm isolates in refrigerated raw milk. Mean colony counts of three experiments are shown at time points 0 d (maximum standard error of the mean ±0.6), 1 d (±0.7), 2 d (±1.0), 3 d (±0.7), 4 d (±1.0), 5 d (±0.3), and 6 d (±0.1). Dashed lines indicate the decrease of *C*. *jejuni* counts below the quantification limit (0 log_10_ CFU/ml): between 0 and 3 d (ST-58 isolates), between 2 and 3 d (ST-50 and ST-45 isolates), between 3 and 4 d (ST-61 isolate and control strain NCTC 11168), and between 4 and 6 d (ST-883 isolates). ST-883 isolate (Cj_Farm3_2014–09_F1) from another dairy farm [11] could be quantified at every time point for 6 d. Summary statistics of the data are presented in Table A in S1 Datasets.

Persistent contamination of bulk tank milk was hypothesized to be due to prolonged survival of the outbreak strain in refrigerated raw milk. Indeed, outbreak type (ST-883) isolates from bulk tank milk (ST883_2013–02_M1), cattle (ST883_2013–01_C7), and milk filters (ST883_2013–01_F1 and ST883_2013–01_F2) survived in milk for four to five days, whereas the other farm isolates survived for only three days or less (Fig 2). Survival for three days was observed for ST-61 isolate and the control strain NCTC 11168. ST-45, ST-50, and one ST-58 isolate survived for two days. Two ST-58 isolates reached the quantification limit already within one day. However, a milk filter isolate (Cj_Farm3_2014–09_F1) of ST-883 from the other Finnish dairy farm survived in milk longest, at least for six days, despite not being detected in bulk tank milk in the longitudinal study [11]. Survival in refrigerated raw milk varied within the lineage ST-21 CC and even within the same ST, indicating unconserved traits behind survivability.

### Aerotolerance

**Fig 3:**
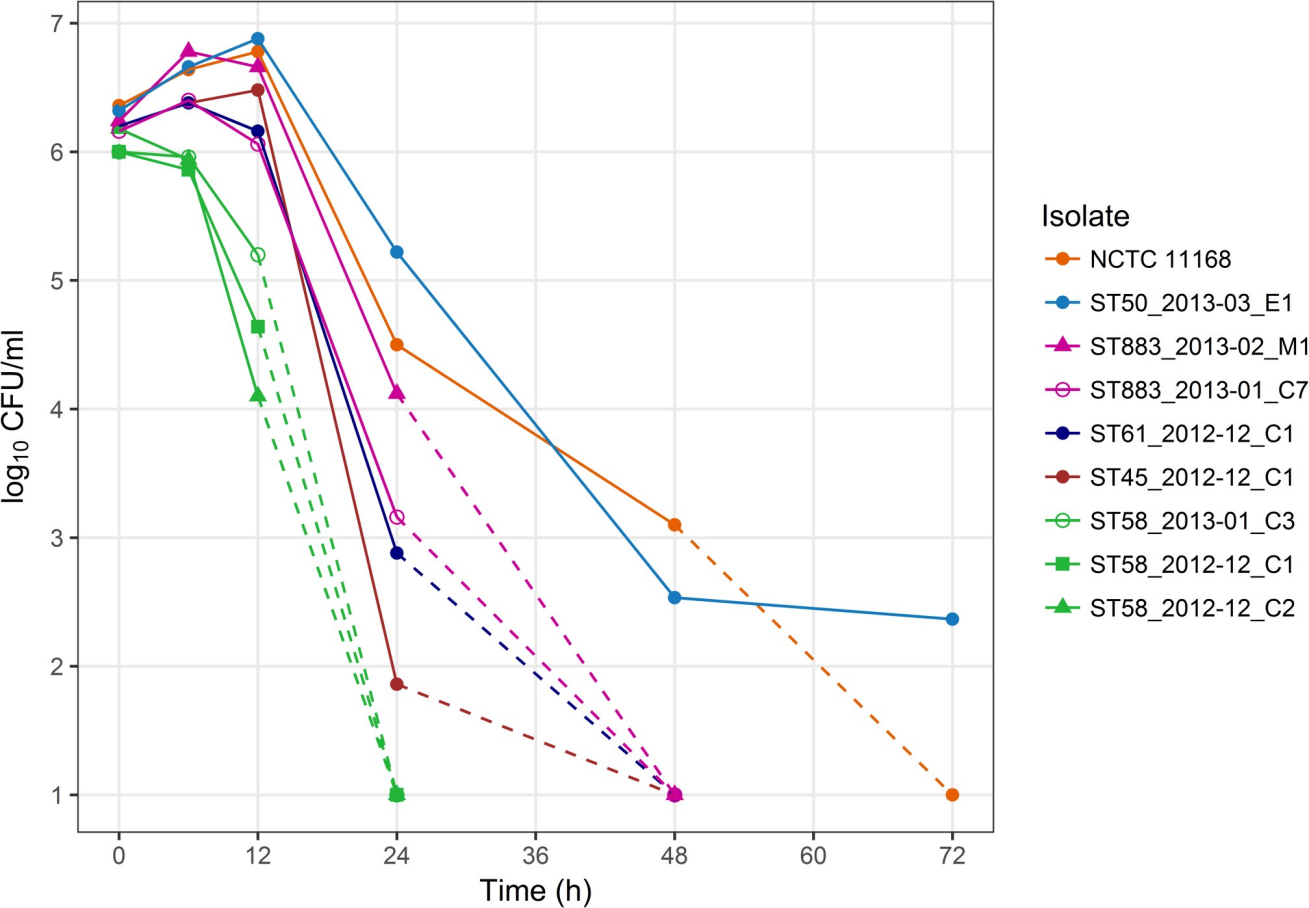
Survival of *C*. *jejuni* farm isolates in broth cultures under aerobic shaking at 41.5°C. Mean colony counts of five experiments are shown at time points 0 h (maximum standard error of the mean ±0.2), 6 h (±0.3), 12 h (±0.5), and 24 h (±1.2) and mean colony counts of three experiments at 48 h (±2.1), and 72 h (±1.4). Dashed lines indicate the decrease of *C*. *jejuni* counts below the quantification limit (1 log_10_ CFU/ml): between 12 and 24 h (ST-58 isolates), between 24 and 48 h (ST-883, ST-61, and ST-45 isolates), and later (ST-50 isolate and control strain NCTC 11168). Summary statistics of the data are presented in Table B in S1 Datasets.

Aerotolerance of *C*. *jejuni* could enhance environmental fitness, thus aiding transmission in the farm environment and survival in bulk tank milk and milk filters. As defined by Oh et al. [12], aerotolerant strains survive after 12 h and hyper-aerotolerant strains after 24 h of aerobic shaking. In our study, all representative farm isolates survived after 12 h of aerobic shaking in five experiments (Fig 3). Survival after 24 h was observed in five experiments for ST-21 CC isolates: ST-883 isolate from bulk tank milk, ST-50 isolate from an udder cloth, and the control strain NCTC 11168. In addition, survival in two or three of five experiments was detected for ST-883, ST-61, and ST-45 cattle isolates after 24 h and survival in one of three experiments for ST-50 and NCTC 11168 after 48 h. Interestingly, isolates from bulk tank milk (outbreak type ST-883) and an udder cloth (ST-50) showed hyper-aerotolerance consistently, as opposed to cattle isolates. Survival under aerobic shaking conditions did not, however, correlate with survival in refrigerated raw milk (Pearson coefficient 0.23, P = 0.56), and other mechanisms were thus suspected to contribute to survival in milk.

### Biofilm formation

Persistence of the outbreak strain in bulk tank milk could possibly also be explained by biofilm formation in the milking machine or milk tank, enhancing the survival and transmission of *Campylobacter*. As an indicator for biofilm, rinsing water of the milking machine was analyzed twice for *Campylobacter* (in May and June 2013). No *Campylobacter* were detected from the water samples, despite simultaneous isolation of *C*. *jejuni* from bulk tank milk.

**Fig 4:**
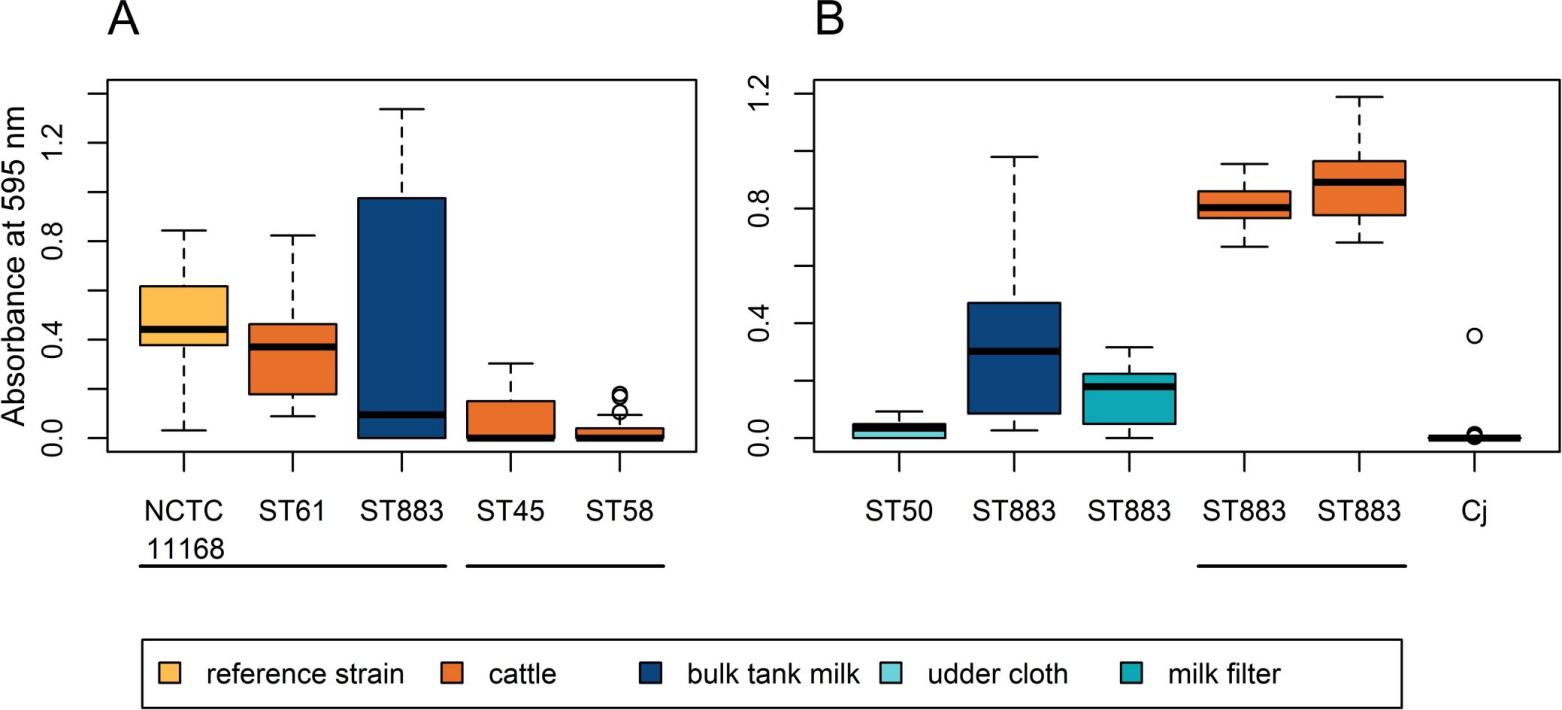
Biofilm formation of *C*. *jejuni* farm isolates on polystyrene microplates during 48-h incubation. Boxplots show median (bold horizontal line), 25% quartile, and 75% quartile biofilm quantities of 18 replicates, indicated by the absorbance of crystal violet stain. Biofilm quantities that fall outside the box by the maximum of 1.5 times the box height (or interquartile range) are shown as whiskers, and the quantities that fall outside the whiskers are shown as circles, indicating possible outliers. Horizontal lines group pairs of means that are not significantly different from each other (t test with no assumption of equal variances on transformed data, P>0.05). Experiment setups A and B are shown respectively in panes A and B. (A) Comparison of the milk isolate (ST883_2013–02_M1) with cattle isolates of other STs. Two ST-58 isolates showed no difference to ST58_2012–12_C1 and are thus omitted from the plot. (B) Comparison of the milk isolate (ST883_2013–02_M1) with ST-50 isolate from an udder cloth and with ST-883 isolates from milk filter (ST883_2013–01_F1) and cattle (ST883_2013–01_C7 and ST883_2012–12_C6), and with ST-883 milk filter isolate (Cj_Farm3_2014–09_F1) from another dairy farm [11], all representing ST-21 CC.

Biofilm formation of representative farm isolates was examined in monocultures on polystyrene microplates. The outbreak strain formed biofilm during 48-h incubation in higher quantities (P≤0.039) than four cattle isolates (Fig 4A). No difference was observed in biofilm quantities (P≥0.14) between the outbreak strain, control strain NCTC 11168, and one cattle isolate (ST-61). Although biofilm formation could not be detected from the milking machine, the outbreak strain was able to form biofilm in laboratory settings.

To further explore whether biofilm formation could explain survival in milk, we studied biofilm formation of the outbreak type (ST-883) isolates from different sample materials (Fig 4B). Indeed, cattle isolates formed biofilm during 48-h incubation in higher quantities (P<2.7×10^−6^) than isolates from milk and milk filters. In addition, more variation in biofilm formation was observed between the replicates of the milk isolate (95% CI: ±0.12) than the replicates of the cattle isolates (95% CI: ±0.07). The milk isolate formed biofilm in an on/off manner between replicate cultures and in higher quantities than the milk filter isolate (P = 0.03). Interestingly, ST-883 milk filter isolate (Cj_Farm3_2014–09_F1) from the other Finnish dairy farm formed no biofilm, suggesting that biofilm formation could contribute to the persistence of the outbreak strain in bulk tank milk.

### Comparative genomics

**Fig 5:**
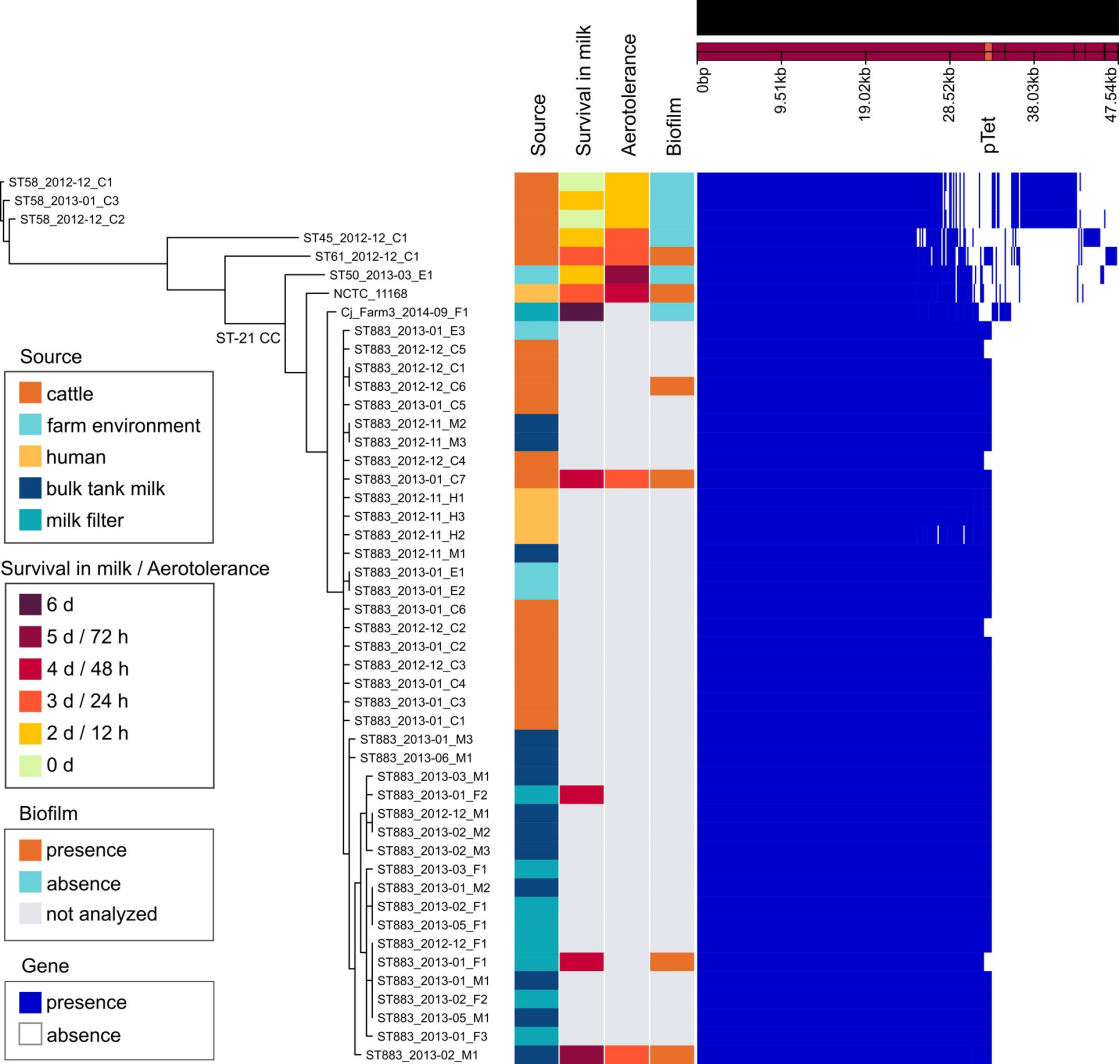
Genomic comparison of *C*. *jejuni* isolates from the outbreak farm. The reference strain NCTC 11168 (RefSeq accession no. NC_002163.1) and ST-883 isolate (Cj_Farm3_2014–09_F1) from another dairy farm [11] are included in the comparison. (A) Left pane: Approximation of maximum-likelihood phylogeny based on the nucleotide alignment of 1356 core genes from Roary using FastTree (version 2.1.9) with the GTR+CAT model [13]. Branches with support <1 are not shown and recombinations are not masked. Names of the farm isolates indicate: ST, sampling time (year-month), sample source (C, E, H, M, or F; see legend), and isolate number. Middle pane: sample source and phenotype. Right pane: presence and absence of genes according to Roary.

**Fig 6:**
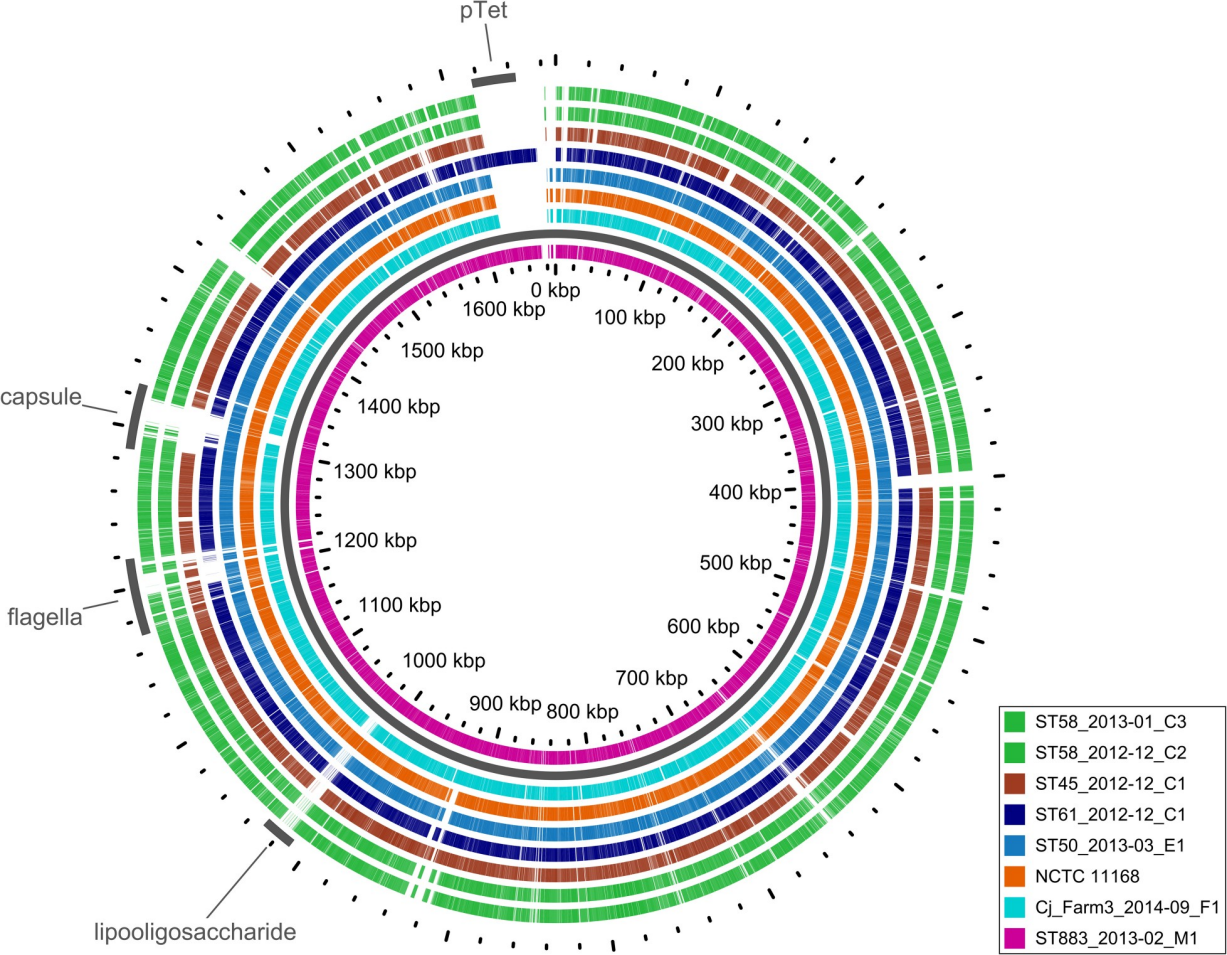
BLAST atlas of the representative farm strains and NCTC 11168 against the outbreak strain (ST883_2013–02_M1).

To recognize potential genotypic features behind the surviving phenotype of the outbreak strain, draft genomes of 46 dairy farm isolates, which represented both the outbreak type (40 isolates) and other STs (6 isolates), were studied for genomic content. Comparison included the reference strain NCTC 11168, representing ST-43, and the milk filter isolate (Cj_Farm3_2014–09_F1) from another Finnish dairy farm, representing ST-883 [11]. Gene content of the outbreak strain closely resembled that of the reference strain NCTC 11168, both representing ST-21 CC (Figs 5 and 6). Of 1620 Prokka-annotated genes of NCTC 11168, the outbreak strain shared 1592 genes (98.2%). Other farm strains shared 97.6% (ST-50) to 88.6% (ST-58) of the reference strain genes. ST-883 isolate from the other dairy farm shared 96.8% of the reference strain genes and 95.0% of the outbreak strain genes (Fig 5).

**Fig 7:**
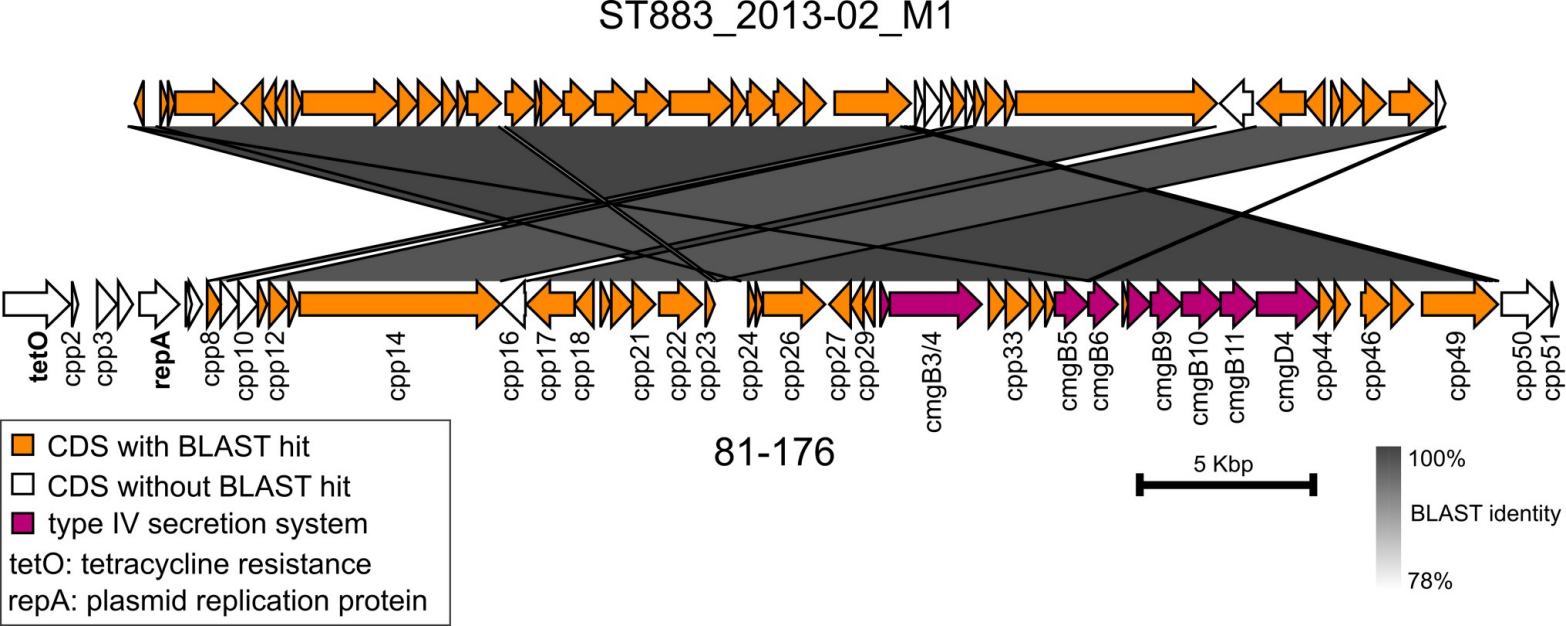
Nucleotide sequence comparison of pTet. The pTet-like element from the outbreak type isolate (ST883_2013–02_M1) is compared against the pTet plasmid from the *C*. *jejuni* strain 81–176 (RefSeq accession no. NC_006135.1). Arrows indicate predicted coding sequences (CDS).

Most strikingly, the outbreak strain harbored a pTet-like element that showed 99.7% nucleotide sequence identity (coverage 93%) with the pTet plasmid of strain 81–176 (Fig 7). The pTet-like element was present in 36 (90%) of the outbreak type isolates and also in the ST-61 cattle isolate with an identical nucleotide sequence, suggesting horizontal transfer between these farm strains (Figs 5 and 6). Compared with the pTet plasmid of strain 81–176, the pTet-like element of the farm strains lacked 12 genes, including genes that encode tetracycline resistance and plasmid replication protein (Fig 7). Concordantly, the farm isolates were susceptible to tetracycline, along with all other tested antimicrobial agents. The pTet-like element harbored 43 predicted genes, including genes that encode the complete type IV secretion system. Six predicted genes were missing from the pTet plasmid of strain 81–176. These genes were annotated to encode hypothetical proteins and resembled (BLAST identity >99.4%, coverage 100%) those in previously sequenced *C*. *jejuni* plasmids.

Excluding the pTet-like element, no unique gene content was detected in the outbreak strain (Fig 6). Opposed to the reference strain NCTC 11168, functional di-/tripeptide transporter (DtpT; locus tag Cj0654c in NCTC 11168, RefSeq accession no. NC_002163.1) was annotated in the outbreak strain, which harbored two adjacent *dtpT* genes, one intact (*dtpT_1*) and one fragmented (*dtpT_2*). Organization of these genes varied among the farm strains: ST-45 strain harbored two intact genes, whereas ST-50 and ST-61 harbored intact *dtpT_1* and fragmented *dtpT_2* gene. ST-883 isolate from the other dairy farm harbored *dtpT* genes identical to the outbreak strain. The presence of *dtpT* genes was further studied within the global genealogy of *C*. *jejuni*, comprising 1159 isolates of non-human origin and 46 isolates from this study (Fig in S1 Appendix). The presence of intact *dtpT_2* was associated with a few clonal complexes: ST-45 CC, ST-283 CC, and ST-42 CC. However, clonal complexes were not associated with *dtpT_1*. Intact *dtpT_1* was most abundant among isolates from ruminants (87.7%), but was frequently observed also among isolates from other host taxa (≥40.0%) (Table in S1 Appendix).

Outbreak type isolates were further studied for genomic adaptation to their isolation source by analyzing single-nucleotide polymorphisms (SNPs), insertions, and deletions. Phase variation was observed in genes related to the capsule, flagella, and oxidative stress response (Table 1). Interestingly, a higher proportion of cattle isolates harbored a phase-variable, fragmented variant of cytochrome C551 peroxidase (Cj0020c) and an intact variant of capsular methyltransferase (Cj1420c) than isolates from milk, suggesting reversible adaptation by oxidative stress response and capsular variation inside or outside the cattle host. Gene organization in the capsular locus of the outbreak strain resembled that of the reference strain NCTC 11168.

**Table 1:** SNPs, insertions, and deletions among the outbreak type isolates (n = 39) against the in-group reference ST883_2013–01_C5, isolated from cattle^1^.

			Location in reference ST883_2013–01_C5			Sequence variation				Proportion (%) of isolates representing an alternate variant by sample source			
Function	Prokka annotation	Locus tag in NCTC 11168 (NC_002163.1)	Locus tag	Contig	Position	Type	Reference	Alternate	Gene fragmentation by variation in	Human (n = 3)	Cattle (n = 12)	Milk (n = 13)	Milk filter or environment (n = 11)
oxidative stress response	cytochrome C551 peroxidase	Cj0020c	KBNHAAAG_01697	14	66849	del	CAAAATTC	CAAATTC	reference	100	25	100	100
-	iron-binding protein	Cj0045c	KBNHAAAG_00019	1	22262	del	TCCCCCCCCCCCATAT	TCCCCCCCCCCATAT	reference	0	17	8	9
-	rod shape-determining protein MreB	Cj0276	KBNHAAAG_00237	3	13418, 13512, 13527, or 13892	snp^2^	G, A, G, or A	A, T, A, or C	-	100	0	8	0
flagella	N-acetyltransferase	Cj1296	KBNHAAAG_01245	8	122950	del	CGGGGGGGGGGAGGTTA	CGGGGGGGGGAGGTTA	alternate	100	25	8	9
	hypothetical protein	Cj1305c	KBNHAAAG_01254	8	129282	del	ACCCCCCCCCCATAA	ACCCCCCCCATAA	reference	67	42	69	45
	hypothetical protein	Cj1306c	KBNHAAAG_01256	8	130522	ins	ACCCCCCCCATA	ACCCCCCCCCATA	reference	0	33	85	36
	hypothetical protein	Cj1318	KBNHAAAG_01269	8	141128	ins	TGGGGGGGGTAT	TGGGGGGGGGGTAT	reference	33	33	31	36
	pseudaminic acid biosynthesis protein PseA	Cj1324	KBNHAAAG_01277	8	146844	ins	TTTAG	TATTAG	alternate	0	33	0	0
	isomerase	Cj1330	KBNHAAAG_01283	8	151640	snp^3^	C	T	-	0	0	69	64
	protein PseD	Cj1335	KBNHAAAG_01288	8	157973	ins	TGGGGGGGGGTAT	TGGGGGGGGGGTAT	reference	0	8	15	9
	hypothetical protein	Cj1342c	KBNHAAAG_01293	9	5364	del	ACCCCCCCCCATA	ACCCCCCCCATA	alternate	0	8	23	45
capsule	methyltransferase	Cj1420c	KBNHAAAG_01367	9	82963	del	ACCCCCCCCCTGT	ACCCCCCCCTGT	alternate	67	8	62	55
	hypothetical protein	Cj1429c	KBNHAAAG_01377	11	6050	del	TCCCCCCCCCCCATTA	TCCCCCCCCCCATTA	reference	33	33	38	55
	aminotransferase	Cj1437c	KBNHAAAG_01385	12	3713	del	TCCCCCCCCCCGCCAGT	TCCCCCCCCCGCCAGT	reference	0	25	8	9
	D-arabinose 5-phosphate isomerase KpsF	Cj1443c	KBNHAAAG_01391	12	12376	snp	G	T	-	0	25	0	9

^1^Only coding loci with variation in four (10%) or more isolates are shown.

^2^Each isolate (n = 4) harbored one SNP in a unique position.

^3^SNP was observed in isolates sampled in January 2013 and thereafter.

## Discussion

This outbreak-related dairy farm study revealed persistent *C*. *jejuni* contamination of bulk tank milk for seven months or longer. The outbreak and the persisting milk contamination were caused by a single clone of sequence type ST-883 (ST-21 CC), which was also prevalent in the dairy herd and shed in feces repeatedly by two cows. In addition to the outbreak clone, five other clones were sporadically detected in the herd (ST-45, ST-58, and ST-61; CCs other than ST-21 CC) and in an udder cloth (ST-50; ST-21 CC), but not in bulk tank milk. They represented both generalist (ST-21 CC and ST-45) and cattle-associated (ST-61 and ST-58) lineages of *C*. *jejuni*. As previously suggested, simultaneous isolation of several *C*. *jejuni* genotypes in livestock-associated environments is common due to their inability to competitively exclude each other [10].

Persistent contamination by *C*. *jejuni* has been reported in bulk tank milk previously. Bianchini et al. [14] noted persistent milk contamination due to subclinical mastitis caused by *C*. *jejuni* (ST-38; ST-48 CC). Mammary excretion of *C*. *jejuni* could not be excluded in our study, as no milk samples were collected directly from cow specimens. However, our study revealed strain-specific characteristics that were potentially linked to survival in milk despite the contamination route. In addition, long-lasting milk contamination by *C*. *jejuni* (ST-50) was previously associated with poorly fitting milking liners, which allowed suction of fecal material-containing air inside the milking machine during milking [15,16]. In our study, rigorous hygienic measures were applied to eliminate milk contamination, and these measures included changing of milking liners. None of these measures showed an effect, and we could not identify the contamination route. Continuous application of hygienic measures yielded low counts of total bacteria (2000–15 000 cells/ml) and somatic cells (115 000–237 000 cells/ml) in milk despite the continued presence of *C*. *jejuni*. Inefficiency of hygienic measures against *C*. *jejuni* persistence in dairy cattle herds was also reported in a longitudinal study [11]. In that study, *C*. *jejuni* ST-883 persisted on a dairy farm for 11 months or longer, but was not isolated from bulk tank milk in weekly samplings. Lack of *C*. *jejuni* isolation from milk in the longitudinal study further raised the question of whether survival in milk, and thus detection in milk, is strain-dependent.

Indeed, survival in refrigerated raw milk varied between and among STs in this study, demonstrating variation between strains. The outbreak type isolates survived for four to five days in refrigerated milk, whereas other isolates from the outbreak farm survived only for three days or less in an experiment with high inocula (10^5^ CFU/ml). With natural detection levels of the outbreak strain observed in this study (0.007–35 MPN/ml), contamination events by poor-surviving, sporadic strains likely remained undetected by the time that the sample shipment reached the laboratory and analyses were initiated, usually within 24 h. However, the ST-883 isolate from the longitudinal study [11] survived in refrigerated milk even longer, at least six days, than the outbreak strain, suggesting that survival in refrigerated raw milk cannot alone explain persistence of the outbreak strain in bulk tank milk.

In both laboratory settings and primary production of milk, milk-contaminating *C*. *jejuni* is exposed to oxygen species. Oxygen species in raw cow’s milk may be produced by the action or metabolism of other microbes (lactic acid bacteria), somatic cells (leukocytes), or antibacterial enzymes (mainly lactoperoxidase) present in milk [17–20]. Atmospheric oxygen is also introduced in milk by stirring. Oxygen tolerance has previously been shown to vary between *C*. *jejuni* isolates, and higher proportions of aerotolerant isolates have been reported among ST-21 CC, which is prevalent in food processing environments and among clinical isolates [12]. Therefore, we hypothesized that enhanced oxygen tolerance of *C*. *jejuni* could contribute to survival in milk or in the farm environment. No correlation was, however, observed between survival under aerobic shaking and in refrigerated raw milk among the farm isolates of this study, and other mechanisms were thus suggested to contribute to survival in milk.

Isolates from milk (ST-883) and an udder cloth (ST-50) showed hyper-aerotolerance consistently, unlike cattle isolates, which could indicate adaptation of *C*. *jejuni* after oxygen exposure in the environment. Concordantly, the majority of the outbreak-type cattle isolates and none of the isolates from other sources, including milk, showed impairment of cytochrome C551 peroxidase gene (Cj0020c) due to phase variation. Phase variation is a rapid adaption mechanism of *C*. *jejuni*, yielding reversible genotypes [21]. *C*. *jejuni* harbors two homologs of cytochrome C551 peroxidase gene, Cj0020c and Cj0358, the former of which has been associated with oxygen stress defense and chick colonization [22,23]. Our results further demonstrate that function of this gene may be reversibly adapted to survival inside and outside the cattle host, respectively, from low to high oxygen tensions.

Although oxygen tolerance did not explain the survival of *C*. *jejuni* in refrigerated raw milk, it may still be a consequence of other adaptation mechanisms such as biofilm formation. In biofilm formation, cells first adhere to a surface, aggregated, and cell metabolism is then adapted to biofilm lifestyle: towards iron uptake, oxidative stress defense, and membrane transport [24]. Indeed, adaption by sample source was observed in biofilm formation of the outbreak type isolates in this study. Cattle isolates formed biofilm in higher quantities than isolates from milk and milk filter in microaerobic conditions at 41.5°C. More variation, appearing in an on/off manner, was observed in biofilm quantities among replicates of the milk isolate than among replicates of the cattle isolates. These results suggest that the milk isolate was reversibly adapted to survival in milk at the cost of biofilm formation or surface adhesion.

The observed on/off variation in biofilm quantities between replicates could be due to phase variation. The outbreak type milk isolate harbored phase-variable, impaired capsular methyltransferase gene (Cj1420c) compared with the cattle isolates harboring an intact gene variant. The capsule has previously been reported to contribute to biofilm formation in ST-45 CC using a genome-wide association approach, although the role of this methyltransferase gene remains unclear [8]. Moreover, cytochrome C551 peroxidase gene (Cj0020c) was associated with biofilm formation in ST-21 CC [8]. Therefore, further experiments should be conducted to elucidate the role of these genes in biofilm formation.

Biofilm-forming strains from the outbreak farm (ST-883 and ST-61) also harbored an identical pTet-like element that lacked genes for tetracycline resistance and replication, suggesting horizontal transfer and replication within the chromosome. The element contained genes that encode a type IV secretion system, which has previously been reported in both plasmid and chromosomal locations in *Campylobacter fetus* species associated with cattle host and to enable conjugative transfer of macromolecules [25]. Type IV secretion system-mediated cell contact has also been suggested to control biofilm formation in *Helicobacter pylori* [26].

In addition to the secretion system, the pTet-like element carried other genes of the pTet plasmid, most of them with unknown function. As no tetracycline resistance gene was present, the element could provide *C*. *jejuni* with another fitness advantage that exceeds the metabolic cost of carrying this genomic element. Four outbreak type isolates (10%) from cattle and a milk filter (ST883_2013–01_F1) had lost the element either on the farm or in the laboratory. All milk isolates harbored the pTet-like element, raising the question of whether this element could contribute to survival in milk or biofilm formation. Outbreak type isolates with and without the pTet-like element survived in refrigerated raw milk equally long, indicating that this element does not affect survival in refrigerated milk. Interestingly, an outbreak type isolate lacking the pTet-like element formed less biofilm than the other outbreak isolates, suggesting that the element may contribute to biofilm formation. Growing evidence from various other pathogenic enterobacteria has also suggested that the presence of plasmids may enhance biofilm formation [27–29]. Further studies should, however, be conducted to understand the mechanisms behind these observations in more detail.

Interestingly, the outbreak strain harbored an intact di-/tripeptide transporter gene (*dtpT*), which shared an identical nucleotide sequence to the ST-883 strain from the other dairy farm [11]. Peptide transporters are essential for the growth of the lactic acid bacterium *Lactococcus lactis* in milk, and DtpT plays a role in peptide-dependent signaling of *L*. *lactis* [30,31]. Furthermore, *Listeria monocytogenes* strains that persisted in the dairy environment showed higher expression levels of *dtpT* gene than non-persisting strains [32]. As gene expression was not investigated in our study, it remains inconclusive whether phenotypic differences arose from differential expression patterns. Within the global genealogy of *C*. *jejuni*, prevalence of the gene *dtpT_1* was highest among *C*. *jejuni* isolates from ruminants, although the gene was also common among isolates from other host taxa. Therefore, it remains to be elucidated whether *dtpT_1* provides a fitness advantage to *C*. *jejuni* in bovine host and farm environments. *C*. *jejuni* relies on amino acids in its energy metabolisms and compensates growth restriction in the host by peptide transport [33]. This provides rationale for further studies on the role of peptides and peptide transporters also in the survival of *C*. *jejuni* in milk.

In conclusion, this study reports persistent contamination of bulk tank milk for seven months or longer, which was recognized during a campylobacteriosis outbreak and was caused by a single *C*. *jejuni* clone of ST-883. Together with previous findings [11], we conclude that ST-883 strains are able to persist on dairy farms and may thus pose a higher health risk in milk production settings than some other *C*. *jejuni* STs. ST-883 strains survived in refrigerated raw milk longer than the other *C*. *jejuni* STs in this study, suggesting that these strains may share some features that promote their survival in milk. Candidate phenotypic and genetic markers were identified here. This study further demonstrates that *C*. *jejuni* may pose a health risk to raw milk consumers despite good on-farm hygiene and emphasizes the importance of avoiding campylobacteriosis by heat treatment of raw milk before consumption.

## Materials and methods

### Campylobacteriosis outbreak

In November 2012, a campylobacteriosis outbreak was recognized among people who had visited or consumed raw drinking milk from a Finnish dairy farm. Two children had been hospitalized with bloody diarrhea and a culture-confirmed *C*. *jejuni* infection. A questionnaire to regular milk-purchasing customers and party-attendees (total response rate 97%) revealed 18/62 (29%) diarrheic respondents, of whom 11/14 (79%) had a culture-confirmed *C*. *jejuni* infection and 12/16 (75%) had drunk raw milk. *C*. *jejuni* was isolated from 7 dairy farm samples (88%), including bulk tank milk, replaceable in-line milk filters of the milking machine, milk room surfaces, and cattle feces. No *C*. *jejuni* was isolated from drinking water in the barn. The *C*. *jejuni* farm isolates were indistinguishable from the patient isolates by PFGE, thus supporting the dairy farm as the source of the outbreak.

### On-farm samplings and hygiene measures

The outbreak-associated dairy farm was located in western Finland and housed 40 cows in pipeline milking. The farm was sampled to trace back the outbreak source in November 2012 and to monitor on-farm hygiene during the following six months. Samplings were conducted by a municipal veterinary officer, entitled by law on foodborne outbreak investigations [34], and thus required no ethics approval. The farm was sampled for bulk tank milk (n = 11) and milk filters (n = 21) weekly during a three-month follow-up (December 2012 to March 2013) and once at six and seven months after the outbreak (May and June 2013). During the follow-up period rigorous on-farm hygienic measures were applied to eliminate *C*. *jejuni* contamination. These measures included acid treatment of the milking machine and milk tank, replacement of milking machine components, and disinfection of the drinking troughs, feeding tables, and stalls.

Samples from cattle feces (n = 39) were taken twice within two months of the outbreak (in December 2012 and January 2013). Five of thirty-three cows were sampled in both samplings. Samples from the farm environment (n = 54) were collected throughout the six-month monitoring period. The samples comprised rinsing water of the milking machine (n = 2), udder cloths (n = 2), and swabs from the milking liners (n = 12), milk room (n = 10), drinking troughs (n = 19), and feeding surfaces (n = 9).

### Sample handling and test portions

Samples were chilled immediately and laboratory analyses were initiated within 24 h of sampling. Bulk tank milk was analyzed as five subsamples of 25 ml. One to four milk filters were collected during the 48 h preceding the milk sampling, refrigerated and moistened with buffered peptone water separately, and analyzed simultaneously with the milk samples. Fecal samples were collected from rectum and analyzed as 10-g test portions. Swab samples were taken with a moistened sponge (Polywipe; Medical Wire and Equipment, Corsham, Wiltshire, UK). A water sample (8 liters) was filtered through 0.45-μm-pore-size membrane filters (GN-6 Metricel Membrane; Pall Corporation, Ann Arbor, MI, USA), and the pooled filters were examined for *Campylobacter*.

### Analysis of farm samples

During outbreak investigation the farm samples were cultured for thermotolerant *Campylobacter* according to NMKL 119:1990 [35] at SeiLab (Seinäjoki, Finland), and *Campylobacter* isolates were further characterized at the national reference laboratory for thermotolerant *Campylobacter* in food, the Finnish Food Authority (Helsinki, Finland). Follow-up samples were cultured for thermotolerant *Campylobacter* at the Finnish Food Authority according to NMKL 119:2007 with the exception of enrichment for 24 h [36]. *Campylobacter* in milk samples were quantified by the most probable number method using three dilutions and five replicates (3×5 MPN) [37]. Species of suspect *Campylobacter* isolates were determined biochemically or by matrix-assisted time-of-flight spectroscopy (MALDI Biotyper, reference library version 4.0.0.1, 5627 main spectra libraries, Bruker Daltonik, Bremen, Germany). Bulk tank milk was monitored for total bacteria and somatic cell counts at the dairy laboratory in separate samplings from this study, using flow cytometry (BactoScan FC; Foss, Hillerød, Denmark) and fluoro-opto-electronic methods [38].

### Subtyping of *C*. *jejuni* isolates

*C*. *jejuni* isolates from each positive sample were subtyped by PFGE with *Sma*I digestion [39]: two or three isolates per milk or milk filter sample and one isolate per fecal or environmental sample. PFGE fingerprints were analyzed using BioNumerics software (version 6.6; Applied Maths, Sint-Martens-Latem, Belgium). Pulsotypes were designated by a difference of one or more bands.

### Whole-genome sequencing, assembly, and multilocus sequence typing

Based on pulsotypes, representative isolates of the outbreak type (n = 40) and other pulsotypes (n = 6) were subjected to whole-genome sequencing. The outbreak type isolates represented patient isolates (n = 3), milk isolates during the outbreak (n = 3), all fecal (n = 13) and environmental isolates (n = 3), and each bulk tank milk (n = 10) and milk filter sampling (n = 8) during the follow-up period of six months.

Paired-end sequencing (read length of 100-bp, 150-bp, or 250-bp) was performed on MiSeq or HiSeq platform (Illumina, San Diego, CA, USA), preceded by the extraction of genomic DNA (PureLink Genomic DNA Mini Kit, Life Technologies, Thermo Fisher Scientific, Carlsbad, CA, USA or DNeasy Blood and Tissue Kit, Qiagen, Hilden, Germany) and preparation of genomic libraries (Nextera XT or Nextera Flex Kit, Illumina). Sequencing reads were subjected to quality control, *de novo* assembly, and MLST using INNUca pipeline (version 4.0.2) (Table C in S1 Datasets) [40]. MLST types were derived from the pubMLST database [41,42]. Within the INNUca pipeline, contamination of reads and assembled contigs were checked with Kraken 2 software against the MiniKraken2_v1_8GB database (retrieved on 13 November 2018) [43,44].

### Whole-genome multilocus sequence typing

Genome assemblies of the farm isolates were further compared by wgMLST with globally isolated genomes of the same MLST ST, using chewbbaca software (version 2.0.16) and INNUENDO schema for *C*. *jejuni* [45,46]. Outbreak type genomes of this study (n = 40) were compared with 137 unique ST-883 genomes available from the INNUENDO database (n = 66), the BIGS database (n = 66; data retrieved on 27 January 2019), and another Finnish dairy farm (n = 5) [11,41,46] (Table D in S1 Datasets). ST-58 genomes of this study (n = 3) were compared with 34 genomes available from the BIGS database (n = 34; data retrieved on 21 March 2019) (Table E in S1 Datasets). After allele calling and extraction of core loci with chewbbaca, minimum spanning trees and distance matrices were calculated using PHYLOViZ Online (http://online.phyloviz.net) and visualized with PHYLOViZ version 2.0, which uses the goeBURST algorithm (Fig 1) [47,48]. Genomes obtained from the BIGS database were regarded as reference material to explore genomic diversity within the farm isolates only.

### Phenotypic characterization

A representative isolate of each pulsotype (n = 8) was selected for phenotypic characterization, including survival in refrigerated raw milk, aerotolerance, biofilm formation, and antimicrobial susceptibility testing. These representatives comprised one isolate of each ST (ST-45, ST-50, and ST-61), all three ST-58 isolates, and two ST-883 isolates (ST883_2013–02_M1 and ST883_2013–01_C7). ST-883 representatives were isolated from milk and cattle, ST-50 from an udder cloth, and other STs from cattle. NCTC 11168 (representing ST-43 and ST-21 CC in MLST) was used as a control strain in all analyses. Additional isolates of the outbreak type (ST-883) from milk filters and cattle were studied for survival in refrigerated raw milk and for biofilm formation, together with an ST-883 isolate (Cj_2014–09_F1) from milk filters of another dairy farm [11].

Isolates were freshly inoculated from glycerol stocks stored at −70°C and, unless stated otherwise, grown under microaerobic conditions (5% oxygen, 10% CO_2_) on ovine blood agar plates at 37°C for 48 h or in Mueller Hinton (MH) broth at 41.5°C without shaking. To obtain inocula of 10^6^ colony-forming units (CFU) /ml for phenotypic analyses, a single colony was inoculated into 10 ml of MH broth, incubated overnight for 16‒18 h, and diluted with fresh broth 10-fold (ST-58 isolates) or 100-fold (other isolates). Colony counts were determined from 1 ml of broth by serial dilutions and plating. All experiments were repeated at least three times, and uninoculated broth was used as a negative control.

### Survival in refrigerated raw milk

Packaged, organic raw milk of the indigenous cattle breed of Finland (Kaskikansa, Saloniemen Juustola, Laitila, Finland) was purchased from retail and stored in aliquots at −70°C on the day of delivery. Overnight cultures were diluted in 100 ml of thawed raw milk to a concentration of 10^5^ CFU/ml in a 250-ml glass flask with a screw cap. Inoculated raw milk was incubated under aerobic conditions at 4°C without shaking. Colony counts were determined on CCDA selective medium (Oxoid, Thermo Fisher Scientific, Wesel, Germany) once a day for six days: after 0 h, 24 h, 48 h, 72 h, 96 h, 120 h, and 144 h of incubation.

### Aerotolerance

Aerotolerance studies were adapted from a protocol presented elsewhere [12]. Overnight cultures were diluted in 10 ml of MH broth to a concentration of 10^6^ CFU/ml. Cultures were incubated under aerobic conditions at 41.5°C with shaking (200 rpm). Colony counts were determined on blood agar plates for three days: after 0 h, 6 h, 12 h, 24 h, 48 h, and 72 h of incubation.

### Biofilm formation

Biofilm formation assay was modified from an earlier protocol [49]. For biofilm formation, plates were incubated at 41.5°C. Overnight cultures were diluted in MH broth to a concentration of 10^6^ CFU/ml, and 75 μl of the culture was transferred to a polystyrene microplate (96 wells, flat bottom, TC-treated, sterile; Corning 3598; Sigma-Aldrich, Germany) in six technical replicates. The microplate was incubated under microaerobic conditions at 41.5°C for 48 h without shaking. The culture broth was then discarded, and the microplate was rinsed with phosphate-buffered saline (PBS), pH 7.4. The microplate was dried at room temperature for 20 min, followed by staining with 100 μl of 1% crystal violet in aqueous solution. After discarding the staining solution and rinsing the microplate with PBS, stained biofilms were eluted with 100 μl of 10% acetic acid and 30% methanol solution (experiment setup A) or 20% acetone and 80% ethanol solution (experiment setup B). Eluted stains were then quantified by measuring absorbance at 595 nm and subtracting the background reading of uninoculated broth.

Subtracted readings were transformed by square root to better meet normality assumption and analyzed using Welch’s analysis of variance for heteroscedastic data. Pairwise two-tailed t tests were then applied with no assumption of equal variances, adjusting P values with the Benjamini-Hochberg method. Data were analyzed in R software (version 3.4.4) [50].

### Antimicrobial susceptibility

Antimicrobial susceptibility was tested by microdilution for erythromycin, ciprofloxacin, tetracycline, streptomycin, gentamicin, and nalidixic acid (VetMIC Camp EU, National Veterinary Institute, Uppsala, Sweden).

### Comparative genomics

Contigs of the genome assemblies were ordered with Mauve (version 2.3.1) against the reference genome NCTC 11168 (RefSeq accession no. NC_002163.1) [21,51,52]. The reference genome was included in further analyses with all 46 genomes from the farm, in addition to the ST-883 isolate from another dairy farm (Cj_Farm3_2014–09_F1) [11]. Genomes were annotated with Prokka (version 1.13) and subjected to pangenome analysis with Roary (version 3.8.0) [53,54]. Roary was run with the option not to split paralogs.

Functional annotations of representative isolates were additionally explored with RAST and SEED Viewer [55]. Geneious (version 10.2.2; Biomatters, Auckland, New Zealand) and Artemis Comparison Tool (version 18.0.2) were used for manual inspection and alignment of genomic regions of interest [56]. BLAST program MegaBLAST was used for nucleotide sequence comparisons [57]. Illustrations were rendered using Phandango (Fig 5), GView (Fig 6), and Easyfig (Fig 7) [58–60]. The following BLAST cut-offs were used for GView: e-value <10^−10^, identity >80%, and length >100 nt and Easyfig: e-value <1.

Nucleotide sequences of suspected plasmid origin were characterized by BLAST comparisons against the NCBI database (accessed 11 June 2019) and against the plasmids of the reference strain 81–176, pTet (RefSeq accession no. NC_006135.1), and pVir (NC_005012.1) [61–64]. The presence or absence of pTet genes was confirmed by mapping reads against the reference.

The presence of *dtpT* genes was studied in 1205 *C*. *jejuni* genomes, comprising 46 genomes from this study and 1159 genomes of non-human origin from the INNUENDO database [46]. The genomes were annotated with Prokka, and Prokka-annotated nucleotide coding sequences were screened against the reference genes from the strain ST45_2012–12_C1 (genes *dtpT_1* and *dtpT_2*; locus tags BELOJCJC_00581 and BELOJCJC_00582) using ABRicate (version 0.9.3) [65]. The reference genes shared nucleotide sequence identity of 64.9% with each other and represented the longest gene variants (1554 and 1530 nt, respectively) among the isolates of this study. The genes were considered present within the queried genomes with gene coverage of 100% and fragmented with coverage below 100%.

Genomes of the outbreak type (n = 40) were further analyzed for SNPs, insertions, and deletions using Snippy (version 3.2-dev) [66]. In the analysis, INNUca-trimmed reads of 39 genomes were mapped against an in-group reference genome (ST883_2013–01_C5), annotated by Prokka. The in-group reference was selected based on the highest number of coding sequences.

## Supporting information

S1 Appendix Presence of genes that encode di-/tripeptide transporter (*dtpT*) within the global genealogy of *C*. *jejuni*. (PDF)

S1 Datasets Summary statistics of survival data, quality control of whole-genome sequences, and metadata for wgMLST analyses. (XLSX)
