# Supplementary material for: Persistent contamination of raw milk by Campylobacter jejuni ST-883
Source: PLoS One. 2020 Apr 21;15(4):e0231810. doi: 10.1371/journal.pone.0231810 (PMC7173850; doi:10.1371/journal.pone.0231810)
Supplement: S1 Appendix — (PDF) [file pone.0231810.s001.pdf]

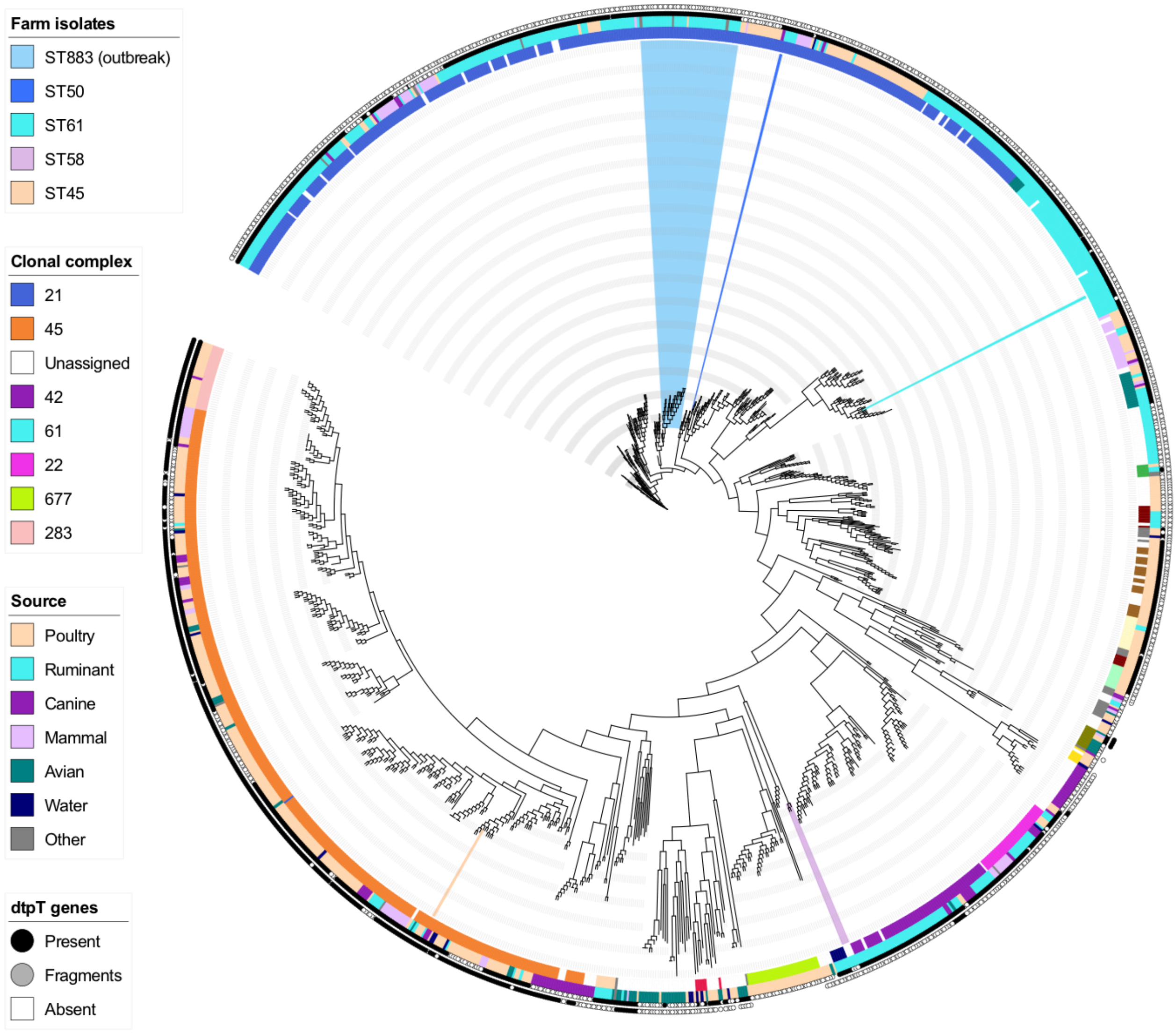

Fig. Presence of genes that encode di-/tripeptide transporter (*dtpT*) within the global genealogy of *C. jejuni*. Core-genome phylogeny of 1205 *C. jejuni* isolates was constructed by aligning 958 core genes using Roary (version 3.8.0) with MAFFT and inferring approximately maximum-likelihood tree using FastTree (version 2.1.9) with the GTR+CAT model [1,2]. The placement of the root is not biologically meaningful, and recombinations are not masked. Farm isolates of this study are highlighted with colored ranges in the tree. Metadata are presented with colored strips and dots in the order starting from the innermost circle: 1. clonal complex, 2. source, 3. *dtpT_1* gene, and 4. *dtpT_2* gene. The tree was visualized using iTOL (version 4.4.2) [3].

Table. Number of *C. jejuni* genomes harboring *dtpT* genes by host among genomes from the INNUENDO database [4].

**Source**	**Total no. of genomes**	***dtpT_1* (%)**	***dtpT_2* (%)**	**Both genes (%)**
Poultry	497	314 (63.2)	233 (74.2)	155 (31.2)
Ruminant	423	371 (87.7)	10 (2.3)	9 (2.1)
Canine	85	34 (40.0)	35 (41.2)	18 (21.2)
Mammal	76	59 (77.6)	33 (43.4)	31 (40.8)
Avian	52	24 (46.2)	19 (36.5)	17 (32.7)

References

[1] Page AJ, Cummins CA, Hunt M, Wong VK, Reuter S, Holden MTG, et al. Roary: rapid large-scale prokaryote pan genome analysis. Bioinformatics 2015 11/15;31(22):3691-3693.

[2] Price MN, Dehal PS, Arkin AP. FastTree 2 – approximately maximum-likelihood trees for large alignments. PLoS One 2010 03/10;5(3):e9490.

[3] Letunic I, Bork P. Interactive Tree Of Life (iTOL) v4: recent updates and new developments. Nucleic Acids Res 2019;47(W1):W256-W259.

[4] Rossi M, Silva MS, Ribeiro-Gonçalves BF, Silva DN, Machado MP, Oleastro M, et al. INNUENDO whole genome and core genome MLST schemas and datasets for *Campylobacter jejuni* (Version 1.0). 2018; In: Zenodo [Internet]. Available from: http://doi.org/10.5281/zenodo.1322564.
